# Multi-finger coordination in healthy subjects and stroke patients: a mathematical modelling approach

**DOI:** 10.1186/1743-0003-8-19

**Published:** 2011-04-20

**Authors:** Ilaria Carpinella, Johanna Jonsdottir, Maurizio Ferrarin

**Affiliations:** 1 Biomedical Technology Department, Found. Don C. Gnocchi Onlus, IRCCS, Via Capecelatro 66, 20148, Milan, Italy; 2 LaRiCE: Gait and Balance Disorders Laboratory, Department of Neurorehabilitation, Found. Don C. Gnocchi Onlus, IRCCS, Via Capecelatro 66, 20148, Milan, Italy

## Abstract

**Background:**

Approximately 60% of stroke survivors experience hand dysfunction limiting execution of daily activities. Several methods have been proposed to objectively quantify fingers' joints range of motion (ROM), while few studies exist about multi-finger coordination during hand movements. The present work analysed this aspect, by providing a complete characterization of spatial and temporal aspects of hand movement, through the mathematical modelling of multi-joint finger motion in healthy subjects and stroke patients.

**Methods:**

Hand opening and closing movements were examined in 12 healthy volunteers and 14 hemiplegic stroke survivors by means of optoelectronic kinematic analysis. The flexion/extension angles of metacarpophalangeal (MCPJ) and proximal interphalangeal joints (IPJ) of all fingers were computed and mathematically characterized by a four-parameter hyperbolic tangent function. Accuracy of the selected model was analysed by means of coefficient of determination (R^2^) and root mean square error (RMSE). Test-retest reliability was quantified by intraclass correlation coefficient (ICC) and test-retest errors. Comparison between performances of healthy controls and stroke subjects were performed by analysing possible differences in parameters describing angular and temporal aspects of hand kinematics and inter-joint, inter-digit coordination.

**Results:**

The angular profiles of hand opening and closing were accurately characterized by the selected model, both in healthy controls and in stroke subjects (R^2 ^> 0.973, RMSE < 2.0°). Test-retest reliability was found to be excellent, with ICC > 0.75 and remarking errors comparable to those obtained with other methods. Comparison with healthy controls revealed that hemiparetic hand movement was impaired not only in joints ROM but also in the temporal aspects of motion: peak velocities were significantly decreased, inter-digit coordination was reduced of more than 50% and inter-joint coordination patterns were highly disrupted. In particular, the stereotypical proximal-to-distal opening sequence (reversed during hand closing) found in healthy subjects, was altered in stroke subjects who showed abnormally high delay between IPJ and MCPJ movement or reversed moving sequences.

**Conclusions:**

The proposed method has proven to be a promising tool for a complete objective characterization of spatial and temporal aspects of hand movement in stroke, providing further information for a more targeted planning of the rehabilitation treatment to each specific patient and for a quantitative assessment of therapy's outcome.

## Background

In the last decade, kinematic analysis of upper limb movements has been increasingly investigated [1,2]. Quantitative characterization of upper limb movements are, indeed, highly required in clinical research and practice, not only to obtain information about pathophysiological aspects of neural control but also to quantify impairment of upper limbs, to plan the appropriate therapeutic approach and to quantify the effectiveness of treatment [3]. This is particularly important in the case of stroke which is the leading cause of disability in the adult worldwide with an estimated incidence of 16 million new cases per year [4]. Approximately 60% of stroke survivors experience upper extremity dysfunction limiting execution of functional activities and independent participation in daily life [5]. Chronic deficits are especially prevalent in the hand, as finger extension is the motor function most likely to be impaired [5].

Within recent years, progress in technology has provided several instruments and methods to objectively quantify hand kinematics [3]. The most common are electrogoniometers [6], instrumented gloves [7], electromagnetic systems [8] and optoelectronic kinematic analysers [9-12]. Some of these methods have been used for the evaluation of anomalies in hand kinematics due to hand injury [9], focal dystonia [13] and stroke [8,11,14]. Most of these studies are mainly focused on the analysis of initial and final position of fingers during a specific movement to evaluate active range of motion, while there is still a lack of studies aimed at analysing temporal aspects of hand motion (i.e. the movement process) and multi-finger coordination that is also highly impaired in people with stroke [15].

Motion coordination among long fingers (index to little finger) has been investigated in healthy subjects during unrestricted flexion/extension movements [16,17] and during object-grasping [18,19]. Analysis of temporal aspects of these multi-joints movements revealed the existence of task-specific motion coordination patterns between metacarpophalangeal joints (MCPJ) and proximal interphalangeal joints (IPJ) of digits 2-5. In particular, a proximal-to-distal sequence (i.e. MCPJ start moving first, followed by IPJ) was noticed during free hand opening [16] and hand opening before cylinder-grasping [18], while a reversed sequence (i.e. IPJ-MCPJ sequence) was found during unrestricted hand closing [16]. Temporal coordination of finger motion during the movement to grasp an object was analysed also by Santello et al [19] in unimpaired individuals. Their results demonstrated a high degree of covariation among the rotations of the MCPJ and IPJ of long fingers. Specifically, all joint of the same type (i.e. MCPJ and IPJ) tended to extend and flex together, simultaneously reaching a maximum excursion.

These results gave additional insight into finger motion control in healthy subjects and provided a useful starting point for the analysis of changes in the patterns of joint motion due to neuromuscular disorders, even though in these studies the role of the thumb was often lacking.

Following these considerations, in the present work a quantitative analysis of unrestricted hand opening and closing movements, with particular attention to inter-joint, inter-finger coordination was performed on a group of healthy subjects and on persons with hemiparesis due to stroke.

The selected task (hand opening and closing) was chosen as it represents the most elemental multi-finger movement and has previously been demonstrated to be a reliable early predictor of recovery of arm function in stroke patients [8,20].

The analysis was performed by using the method proposed by Braido & Zhang [18], based on the mathematical characterization of fingers joint motion. This specific method was chosen since the parametric modelling of hand kinematics can provide a synthetic representation of actual movements and facilitate the extraction of spatial, temporal and coordinative features of motion, not immediately computable from measured data.

With respect to the study of Braido & Zhang [18], which reported results related to healthy subjects only and didn't consider the role of the thumb, the present work had three main purposes: i) evaluation of the accuracy of the chosen method in characterizing hand opening/closing movements, including thumb motion, in healthy subjects and persons with hemiparesis due to stroke, ii) evaluation of the method's capacity to discriminate motor performances of stroke subjects from that of healthy controls and iii) analysis of the repeatability of the method, and thus, the minimal detectable change in hand performance that could potentially be used in future work to monitor the progression of hand function in each stroke subject.

## Methods

### Subjects

Twelve healthy volunteers (2 women and 10 men, mean age: 36.6 ± 10.8 years), with no history of injury or surgery to the hand, and fourteen subjects with hemiparesis caused by stroke (7 women and 7 men, mean age: 58.4 ± 14.8 years) participated in the study. All hemiplegic patients had sustained a single ischemic (8 subjects) or hemorrhagic (6 subjects) stroke from 3.5 months to 7.5 years before the experiments. Three subjects had right hemiparesis and eleven had left hemiparesis. All stroke subjects showed a clinically significant reduction of the paretic upper limb function as indicated by the Action Research Arm Test [21] scores ranging from 5 to 46 points (maximum score of 57 points indicates a normal upper limb function). Demographic and clinical data are presented in Table 1. Exclusion criteria were: coexistence of orthopedic, neurological or other medical conditions that limited the affected upper limb, inability to bring the affected hand to the mouth, inability to extend the paretic elbow to at least 120°, spasticity of hand muscles rated more than 3 points on the Ashworth scale [22], botulinum toxin injections in the upper extremity musculature in the last three months, presence of severe hemispatial neglect, aphasia and/or hemianopsia.

**Table 1:** Demographic and clinical data of stroke subjects.

Subject	Age [years]	Gender	Stroke Type	Time after stroke [months]	Side of hemiparesis	ARAT score [points]
ST1	77	M	ISC	80.0	RX	9
ST2	72	F	ISC	36.8	LX	10
ST3	45	F	HEM	90.6	RX	36
ST4	39	M	HEM	78.2	LX	28
ST5	64	F	ISC	3.7	LX	6
ST6	33	F	HEM	8.4	LX	10
ST7	82	F	HEM	8.5	LX	10
ST8	64	M	ISC	37.5	LX	5
ST9	63	F	ISC	48.0	LX	46
ST10	70	M	HEM	58.8	LX	38
ST11	54	M	ISC	8.7	LX	10
ST12	57	M	ISC	3.5	LX	32
ST13	56	F	ISC	10.9	LX	39
ST14	41	M	HEM	4.6	RX	9
						
**Mean**	**58.4**	**7M/7F**	**8ISC/6HEM**	**34.2**	**3RX/11LX**	**20.6**
**SD**	**14.8**			**32.0**		**14.9**

ARAT: Action Reasearch Arm Test

ISC: ischemic stroke

HEM: hemorrhagic stroke

All subjects had given written, informed consent to the experimental protocol, which was conformed to the standards for human experiments set by the Declaration of Helsinki (last modified in 2004) and approved by the local ethical committee.

### Experimental protocol

**Figure 1:**
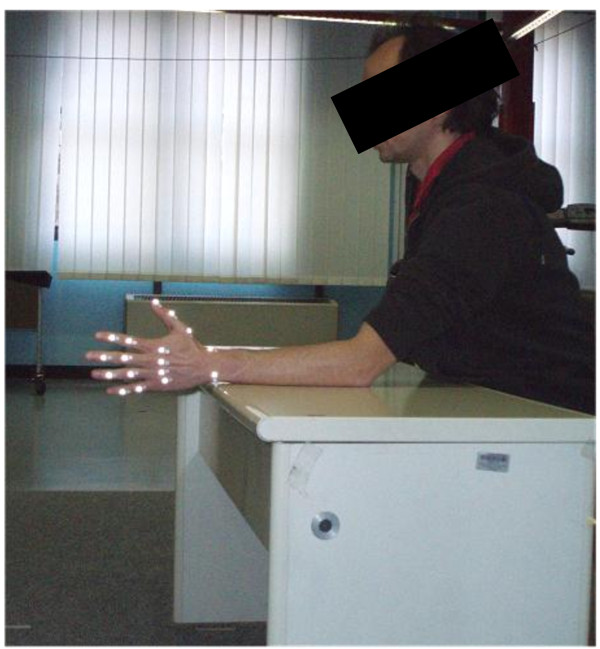
**Experimental set-up**. Example of a subject performing hand opening/closing task.

Subjects were asked to sit upright in a chair behind a table. The forearm was maintained semi-prone on the table, the elbow was flexed of about 120° while the wrist was kept in a neutral position (see Figure 1). Healthy subjects were required to maintain the hand relaxed for 2-3 seconds, open the hand at self-selected speed, rest with the hand maximally opened for 2 seconds, close the hand at self-selected speed and rest with the hand maximally closed for 2 seconds. The sequence was repeated 5 times. Both hands were tested (*Nco = 24*). Subjects with stroke performed, with the paretic hand (*Nst = 14*), the same task but with a resting period of at least 10 seconds between two sequences of hand opening/closing, in order to reduce the effect of fatigue and to minimize the onset of co-contractions [23].

In order to analyse test-retest variations in hand kinematics, all healthy subjects were tested a second time after markers repositioning. A random hand of each subject was evaluated following the same experimental protocol described above.

### Experimental set-up and data pre-processing

**Figure 2:**
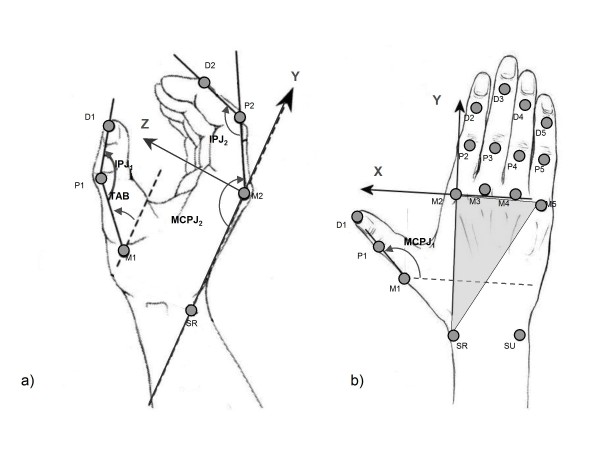
**Marker placement, hand local reference system and finger joint angles**. Markers position. M*i*: head of the metacarpal bone of finger *i *(*i *= 1-5); P*i*: head of proximal phalanx of finger *i *(*i *= 1-5); D*i*: head of distal phalanx of the thumb (*i *= 1) and head of middle phalanx of long fingers (*i *= 2-5); SU: styloid process of ulna; SR: styloid process of radius. Local reference system XYZ. The origin is in correspondence of the marker M2. Vectors (M2-M5) and (M2 - SR) define the metacarpal plane of the hand (grey triangle). Z-axis is normal to the metacarpal plane pointing palmarly, Y-axis has the direction of vector (M2 - SR) pointing distally, while X-axis is calculated as the cross-product of Y and Z-axis, pointing radially. Joint angles in transverse plane YZ (a) and in sagittal plane XY (b) of the hand. MCPJ_*i*_: metacarpophalangeal joint flexion angle of finger *i *(*i *= 1-5); IPJ_*i*_: proximal interphalangeal joint flexion angle of finger *i *(*i *= 1-5); TAB: thumb abduction angle. MCPJ_*i *_(*i *= 2-5) is defined as the angle between Y-axis and the projection of the vector (P*i *- M*i*) on the YZ plane; IPJ_*i *_(*i *= 2-5) is the angle between the projections of vectors (D*i*-P*i*) and (P*i*-M*i*) on the YZ plane. TAB is the angle between the vector (P1 - M1) and the XY plane. MCPJ_*1 *_is the angle between X-axis and the projection of vector (P1 - M1) on the XY plane. IPJ_*1 *_is the angle between vectors (D1 - P1) and (P1 - M1).

Hand kinematics were recorded by an optoelectronic motion analysis system (Smart, EMotion, Italy) consisting of nine infrared video cameras (sampling rate = 60 Hz). The working volume (70 × 70 × 70 cm^3^) was calibrated to provide an accuracy of less than 0.3 mm. Seventeen retro-reflective hemispheric markers, with diameter of 6 mm were attached to the hand of the subjects, according to the protocol described in Carpinella et al.[11], on the bony landmarks shown in Figure 2. After the acquisition, marker coordinates were low-pass filtered using a 5th order, zero-lag, Butterworth digital filter, with a cut-off frequency of 6 Hz.

### Data processing

All data processing and analysis procedures were implemented using MATLAB^® ^software (The MathWorks, Inc., Natick, MA).

#### Joint angle calculation and normalization

A local Cartesian coordinate system XYZ was established, following the procedure described in [11] and the time-courses of the following joint angles computed: metacarpophalangeal joint (MCPJ*i*) flexion/extension angles, proximal interphalangeal joint (IPJ*i*) flexion/extension angles of finger *i *(*i *= 1-5) and thumb abduction angle (TAB) (see Figure 2 for more details). An automatic algorithm was established to identify the initiation and termination of hand opening and closing separately. The initiation time of hand opening/closing (T_start_) was defined as the instant in which the first joint reached an angular velocity value equal to 10% of its own peak velocity (V_pk_), while movement termination (T_end_) was defined as the instant in which the angular velocity of the last joint fell below the 10% of V_pk_. Thereafter, angular profiles were segmented in separated movements of hand opening and closing and normalized in time as a percentage of the movement duration (%Dur).

#### Joint angle mathematical characterization and accuracy

**Figure 3:**
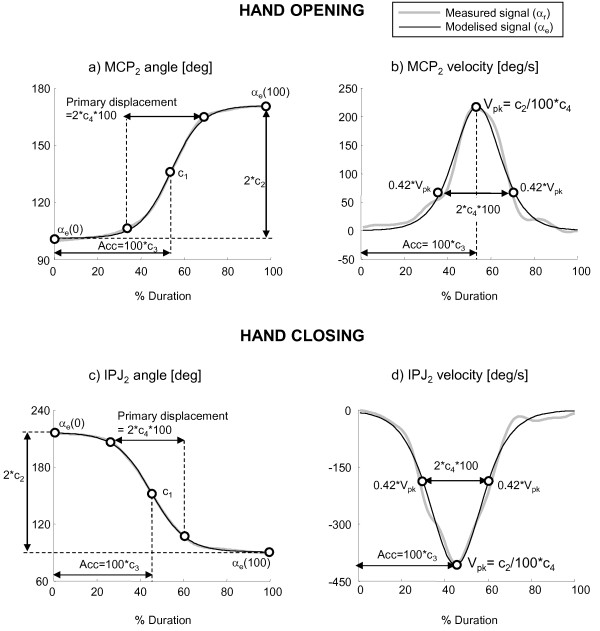
**Measured and estimated signals**. Examples of joint angles and velocity during hand opening (a, b) and hand closing (c, d). Measured signals (grey line) and signals estimated with a four-parameter hyperbolic tangent function (black line) are plotted together. The kinematic meaning of all four parameters is shown.

After data normalization, each joint angular profile was mathematically characterized to obtain a synthetic representation of motion and facilitate the extraction of spatial, temporal and coordinative features of multi-finger movements. The chosen mathematical model was a hyperbolic tangent function with four parameters as suggested in [18,24]. The function, graphically represented in Figure 3, is described by Equation 1:(1)

where *α*_*e*_*(t) *represents the estimated value of a specific joint angle *α*_*r*_*(t) *at instant *t *(*t *= 0,..., *ΔT*), *ΔT = T*_*end*_*-T*_*start *_is the total opening/closing movement duration, *c*_*1 *_= [*α*_*e*_*(*0*)+ α*_*e*_*(ΔT)*]*/*2 is the average of the initial and final angles, *c*_*2 *_= [*α*_*e*_*(ΔT)- α*_*e*_*(0)*]*/*[tanh((1-c_*3*_)/c_*4*_) + tanh (c_*3*_/c_*4*_)] approximates a half of the total angular displacement (i.e. [*α*_*e*_*(ΔT)- α*_*e*_*(0)*]*/*2) when c_*4 *_is sufficiently small with respect to c_*3 *_(e.g. c_*4 *_< = 0.5* c_*3*_)^1^, *c*_*3 *_represents the acceleration portion of the total movement duration and *c*_*4 *_corresponds to the half of the primary displacement time, where the primary displacement is considered the steepest ascending or descending portion of the signal characterized by a velocity (*V*) higher than 42% of peak speed (*V*_*pk*_) [18], as shown by Equations 2 and represented in Figure 3.(2)

A non-linear least square curve fitting approach was used to obtain the set of four parameters that best fit each joint angle profile. The initial estimate of the four parameters were set according to [24]: *c*_*1 *_= [*α*_*r*_*(*0*)+ α*_*r*_*(ΔT)*]*/*2, *c*_*2 *_= [*α*_*r*_*(ΔT) - α*_*r*_*(*0*)*]*/*2, *c*_*3 *_= 0.5 and *c*_*4 *_= 0.25.

To analyse the accuracy of the model, the coefficient of determination (R^2^) and the root mean square error (RMSE) were computed. An angular profile was considered well fitted by the model and included in the subsequent group analysis if R^2 ^was greater than 0.8. Values of R^2 ^below this threshold would suggest that the corresponding joint motion didn't show a sygmoidal-shape profile and for this reason were treated separately.

#### Test-retest reliability

To analyse the test-retest variations on the four parameters c_1_, c_2_, c_3_, c_4_, data from the 12 healthy subjects tested two times for reliability purposes were considered. Test-retest reliability was statistically evaluated using intraclass correlation coefficient, model 2,1 (ICC_2,1_) calculated following the procedure described by McGraw & Wong [25]. ICC_2,1 _is represented by Equation 3:(3)

where σ_n_^2 ^is the inter-subject variance, σ_s_^2 ^is the inter-session variance and σ_r_^2 ^is the intra-session variance. The following guidelines were used to grade the strength of reliability: 0.50-0.60 fair, 0.60-0.75 good, 0.75-1.00 excellent reliability [12,26]. Within-subject variability (σ_w_) was evaluated by the Standard Error of Measurement (SEM), computed, from Equation 3, as √(*σ*_*s*_^*2*^*+σ*_*r*_^*2*^*)*. The percentage ratio between intra-session standard deviation (σ_r_) and within-subject standard deviation (σ_w_) was also computed. For all angular profiles and for each parameter, the absolute difference between the values obtained from the two sessions was computed (absolute test-retest error). Maximum test-retest error and, thus, minimum significant change detectable by the protocol was calculated as *mean absolute error + 2 standard deviations*, following the principles of Bland-Altman analysis [27].

#### Extraction of specific parameters

From data included in the group analysis (R^2 ^> 0.8), the following variables were calculated to analyse three distinct aspects of hand motion:

1) Finger kinematics were analysed through the following parameters:

• Dur = T_end _-T_start_, movement duration

• α_min _= c_1_-c_2_, angle of maximum flexion

• α_max _= c_1_+c_2_, angle of maximum extension

• ROM = 2*c_2_, range of motion

• V_pk _= c_2_/100*c_4_, peak velocity

2) Inter-joint coordination was inspected by looking at the level of synchronization between MCPJ and IPJ, which was defined by the temporal delay (Δ_*i*_) between IPJ and MCPJ angles of finger *i *in the instant of peak velocity (100*c_3_). The value of Δ_*i *_was calculated as 100*[c_3_(IPJ_*i*_)-c_3_(MCPJ_*i*_)].

3) Inter-digit coordination was evaluated considering the variability among IPJ-MCPJ delays (Δ_*i*_) of all fingers: a high level of inter-digit coordination is represented by similar values of Δ_*i *_(low variability), while poor coordination is implied by higher differences among Δ_*i *_(high variability). This concept was represented by the coordination index among long fingers (COI_LF_) and among all digits (COI_HAND_). COI_LF _was defined as 100*CV_LF_(*co*)/CV_LF_(*j*), where CV_LF_(*j*) = standard deviation(Δ_*2*_, Δ_*3*_, Δ_*4*_, Δ_*5*_)/mean(Δ_*2*_, Δ_*3*_, Δ_*4*_, Δ_*5*_) was the coefficient of variation for long fingers of hand *j *and CV_LF_(*co*) was the mean CV_LF _value of healthy control subjects. COI_HAND _was calculated in the same way but considering the coefficient of variations among all 5 fingers. COI values below 100% indicated lower coordination with respect to the mean value of control subjects, while values above 100% represent a level of coordination higher than the average value of healthy subjects.

Data not well fitted by the selected model (R^2 ^< 0.8) were treated separately and only α_min_, α_max _and ROM, as calculated from the measured data, were included in the analysis.

### Statistical analysis

Considering the small sample of data, comparisons were made using nonparametric tests. Differences between IPJ and MCPJ were analysed using Wilcoxon matched pairs test (Wt), variations among fingers were evaluated with Friedman test (Ft) and Bonferroni post-hoc comparisons, while differences between healthy controls and stroke subjects were analysed by means of Mann-Whitney U test (MWt). Level of significance was set to 0.05.

## Results

### Model accuracy

**Figure 4:**
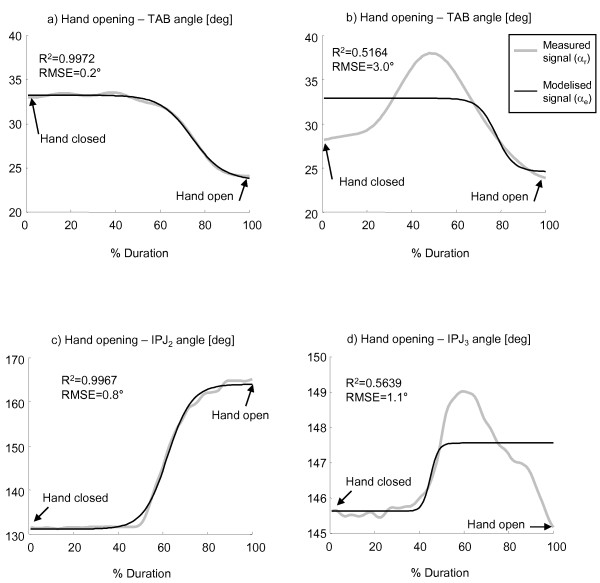
**Examples of measured and estimated angles during hand opening**. Thumb abduction angles (TAB) of two unimpaired individuals (a, b) and proximal interphalangeal joint angles (IPJ_2 _and IPJ_3_) of two stroke subjects (c, d) during movements of hand opening. Coefficient of determination (R^2^) and root mean square error (RMSE) are reported.

Analysis of all hand opening/closing movements performed by healthy subjects confirmed that the selected mathematical model accurately characterized the shape of angular profiles of MCPJ and IPJ of long fingers and thumb. This was confirmed by R^2 ^and RMSE mean (± SD) values which were, respectively, 0.996 (± 0.009) and 1.6° (± 0.6°) for hand opening and 0.995 (± 0.009) and 1.7°(± 0.7°) for hand closing. With regard to thumb abduction angles (TAB), the mathematical model accurately characterised TAB only in 75% of all tested hands (R^2 ^= 0.964 ± 0.043, RMSE = 0.9° ± 0.5°), as shown in Figure 4a. The remaining thumb abduction angles (25%) showed significantly lower values of R^2 ^(0.517 ± 0.210) and higher RMSE (2.6° ± 1.3°), as indicated in the example of Figure 4b. For this reason, TAB angles were considered not well fitted by the selected model and, consequently, only the angular values reached at maximally closed and open hand, as calculated from the measured data, were included in the analysis.

Concerning stroke subjects, 5% of all MCPJ and IPJ angular profiles during hand opening did not show a sygmoidal-shape profile, as indicated by R^2 ^values lower than 0.8 (see Figure 4d). The remaining data (95%) were accurately characterized by the mathematical model as they showed values of R^2 ^and RMSE equal to 0.973 (± 0.045) and 0.9° (± 0.7°), respectively (see Figure 4c). As for hand closing, all angular profiles were well fitted by the hyperbolic tangent model (R^2 ^= 0.979 ± 0.064, RMSE = 2.0° ± 1.3°). The mathematical model accurately characterised TAB only in 75% of all tested hands (R^2 ^= 0.951 ± 0.050, RMSE = 1.0° ± 0.8°). The remaining thumb abduction angles (25%) showed significantly lower values of R^2 ^(0.549 ± 0.193) and higher RMSE (2.2° ± 1.0°). Consequently, only the angular values reached at maximally closed and open hand, as calculated from the measured data, were included in the analysis.

### Test-retest reliability

Results of the test-retest analysis are reported in Table 2. All four parameters showed good to excellent reliability in both hand opening and closing as indicated by mean ICC values greater than 0.75 [12,26]. Mean Standard Error of Measurement (σ_w_) was lower than 5.0° for angular parameters (c_1_, c_2_) and lower than 7.1%Dur for temporal parameters (c_3_, c_4_). Angular parameters (c_1_, c_2_) showed a mean and a maximum test-retest errors lower than 3.1° and 7.2°, respectively, while mean and maximum test-retest errors for temporal parameters (c_3_, c_4_) were lower than 3.6%Dur and 9.0%Dur. Results on the σ_r_/σ_w_% ratio, revealed that less than 10% of within-subject variations (σ_w_) was due to inter-session variability (σ_s_) while more than 90% was due to intra-session variations (σ_r_).

**Table 2:** Mean (SD) values of test-retest parameters

	Hand opening				Hand closing			
	**c_1_**	**c_2_**	**c_3_**	**c_4_**	**c_1_**	**c_2_**	**c_3_**	**c_4_**

ICC_2,1_	0.96 (0.03)	0.88 (0.07)	0.78 (0.06)	0.79 (0.07)	0.96 (0.03)	0.89 (0.04)	0.86 (0.03)	0.84 (0.07)

σ_w_	4.5° (1.4°)	3.8° (1.4°)	6.7%Dur (2.1%Dur)	7.0%Dur (3.1%Dur)	4.3° (1.1°)	5.0° (1.9°)	3.5%Dur (2.0%Dur)	7.1%Dur (2.4%Dur)

σ_r_/σ_w_%	90.8 (4.8)	93.3 (5.9)	98.0 (3.5)	97.6 (4.0)	94.3 (5.6)	99.7 (0.6)	93.4 (5.9)	98.6 (2.0)

Mean test-retest error	2.5° (1.6°)	2.7° (1.9°)	3.6%Dur (2.6%Dur)	2.7%Dur (2.5%Dur)	3.1° (1.9°)	2.8° (2.2°)	3.4%Dur (2.4%Dur)	3.1%Dur (2.9%Dur)

Max. test-retest error	5.7°	6.5°	8.8%Dur	7.7%Dur	6.9°	7.2°	8.2%Dur	9.0%Dur

ICC_2,1_: Intraclass Correlation Coefficient, model 2,1

σ_w_: within-subject standard deviation

σ_r_/σ_w_%: percentage ratio between intra-session standard deviation and within-subject standard deviation

### Hand motion characterization in healthy subjects

#### Fingers kinematics

Healthy controls took 0.9 (± 0.6) seconds to completely open and close the hand. Table 3 reports the results related to the angular variables extracted from MCPJ and IPJ motion of long fingers and thumb. IPJ showed a significantly higher ROM with respect to MCPJ. This was due to a significantly higher maximum flexion angle of IPJ (α_min_= 80.4° ± 7.7°) with respect to MCPJ (α_min _= 96.6° ± 11.2°), when the hand was completely closed. Contrarily, maximum extension angles, corresponding to the position of maximum hand aperture, were similar for both types of joints (MCPJ: α_max _= 186.7° ± 8.1°; IPJ: α_max _= 189.5° ± 8.7°; p(Wt) = 0.2301, n.s.). As reported in Table 3, IPJ revealed a higher peak velocity with respect to MCPJ both in hand opening and closing. IPJ peak speed was similar in the two movements, while MCPJ speed was significantly lower during extension than during flexion.

**Table 3:** Mean (SD) values of the parameters describing hand movement

	CONTROL				STROKE			
	
	Thumb		Long Fingers		Thumb		Long Fingers	
	
	MCPJ	IPJ	MCPJ	IPJ	MCPJ	IPJ	MCPJ	IPJ
ROM [deg]	61.1 (23.8)	64.0 (24.4)	90.3 (12.7)	109.1 (12.5)	25.8*** (18.4)	40.5** (20.0)	50.4*** (20.4)	64.5*** (27.7)
			*§§§*		*§§*		*§*	

Max. ext. angle [deg]	143.0 (14.6)	188.8 (16.8)	186.7 (8.1)	189.5 (8.7)	116.6*** (22.6)	180.4 (18.2)	166.7*** (17.0)	159.5*** (26.8)
	*§§§*				*§§§*			

Max. Flex. Angle [deg]	81.7 (16.4)	125.2 (20.5)	96.6 (11.2)	80.4 (7.7)	90.7 (17.5)	139.6* (18.8)	116.3*** (13.1)	95.0*** (12.8)
	*§§§*		*§§§*		*§§§*		*§§*	

V_pk _- Hand Opening [deg/s]	191.5 (98.8)	272.8 (146.5)	354.2 (181.1)	490.4 (228.4)	22.7*** (23.6)	16.5*** (23.5)	34.6*** (20.8)	40.7*** (30.9)
	*§§*		*§§§*					

V_pk _- Hand Closing [deg/s]	184.1 (109.4)	189.3 (103.4)	279.2 (146.7)	437.7 (172.5)	50.4*** (48.9)	41.5*** (35.4)	51.7*** (31.8)	83.5*** (55.1)
			*§§§*				*§§*	

*p < 0.05, **p < 0.01, ***p < 0.001 (STROKE vs CONTROL, Mann-Whitney U test).

^§^p < 0.05, ^§§^p < 0.01, ^§§§^p < 0.001 (MCPJ vs IPJ, Wilcoxon matched pairs test).

#### Inter-joint and inter-digit coordination

**Figure 5:**
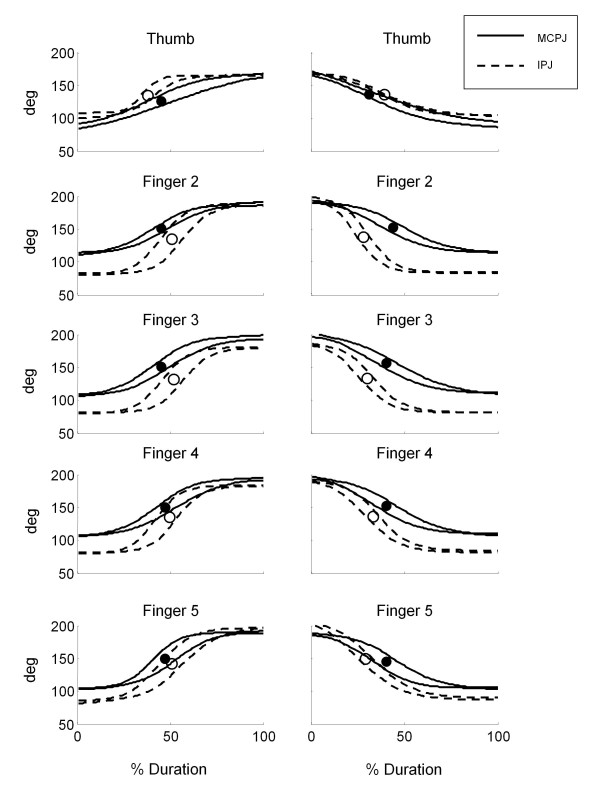
**Example from a healthy subject**. Joint angles (± SD band) of a representative healthy subject during hand opening (left panels) and hand closing (right panels). Instants of peak velocity are represented as black and white dots, for MCPJ and IPJ respectively.

**Figure 6:**
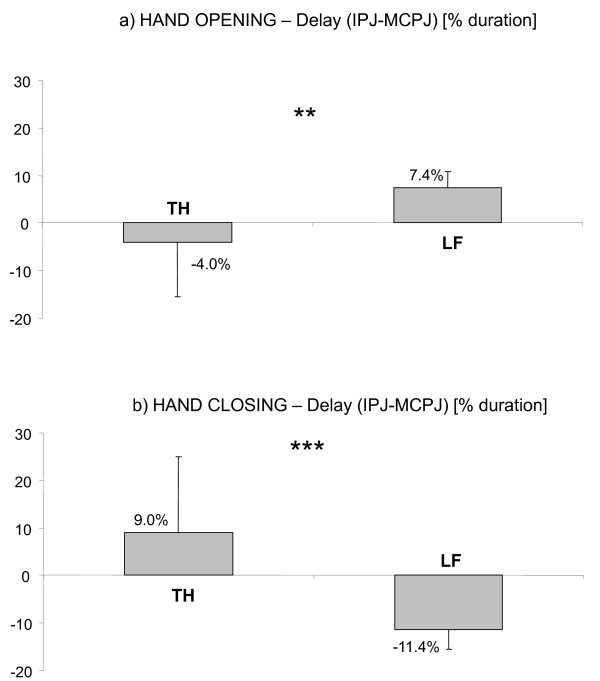
**Inter-joint coordination in healthy subjects**. Results related to the delay between IPJ and MCPJ of thumb (TH) and long fingers (LF) for healthy subjects, during hand opening (a) and hand closing (b). Columns and whiskers represent mean and standard deviation, respectively. **p < 0.01, ***p < 0.001 (TH vs LF, Wilcoxon matched pairs test).

Within each long finger, a proximal-to-distal sequence was evident for hand opening movements (see Figure 5, left panels). In particular, MCPJ started extending first, followed by IPJ after an average delay of 7.4%Dur (see Figure 6a). Contrarily to long fingers, a distal-to-proximal sequence was noticed in the thumb (see Figure 5, upper-left panel): IPJ started extending first followed by MCPJ after a mean delay of 4% (Figure 6a). During hand closing inter-joint sequence was reversed for both thumb and long fingers (see Figure 5, right panels). In particular, a proximal-to distal sequence (i.e. MCPJ-IPJ) was noticed in the thumb and a distal-to proximal sequence (i.e. IPJ-MCPJ) was evident in long fingers (see Figure 6b). In both hand opening and closing MCPJ of finger 2 to 5 moved together, simultaneously reaching peak velocity at approximately 50% of the movement duration. Synchronous motion was noticed also in IPJ, which reached the maximum speed at nearly 57% of the whole duration (see Figure 5).

These coordination sequences were consistent among fingers. In fact, analysis of IPJ-MCPJ delay did not reveal any significant difference among long fingers in hand opening [p(Ft) = 0.2308 n.s.] or closing [p(Ft) = 0.6065 n.s.] indicating a high level of inter-digit coordination.

### Hand motion characterization in subjects with stroke

#### Fingers kinematics

In both hand opening and closing, stroke patients (ST) took significantly longer time to complete the movement with respect to healthy control subjects (CO) (Hand opening: ST = 3.9 s ± 1.7 s, CO = 0.9 s ± 0.6 s, p(MWt) < 0.001; Hand closing: ST = 5.1 s ± 1.6 s, CO = 1.0 s ± 0.6 s, p(MWt) < 0.001). Stroke patients showed a significantly reduced ROM of thumb and long fingers joints that was due to a high reduction of both maximum flexion and maximum extension angles (see Table 3). In three cases, subject's attempt to extend IPJ resulted in an undesired flexion of one or two fingers. No significant differences between controls and stroke subjects were noticed in thumb abduction angles neither in hand opening (ST: 20.1° ± 18.7°, CO: 18.5° ± 17.3°, p(MWt) = 0.5251, n.s.) nor hand closing (ST: 29.7° ± 15.9°, CO: 27.0° ± 11.2°, p(MWt) = 0.5450 n.s.). As reported in Table 3, stroke subjects showed significantly reduced peak velocities in all joints with respect to controls. Moreover, peak speed during hand opening was significantly lower than that obtained during hand closing (p(Wt) < 0.01).

**Figure 7:**
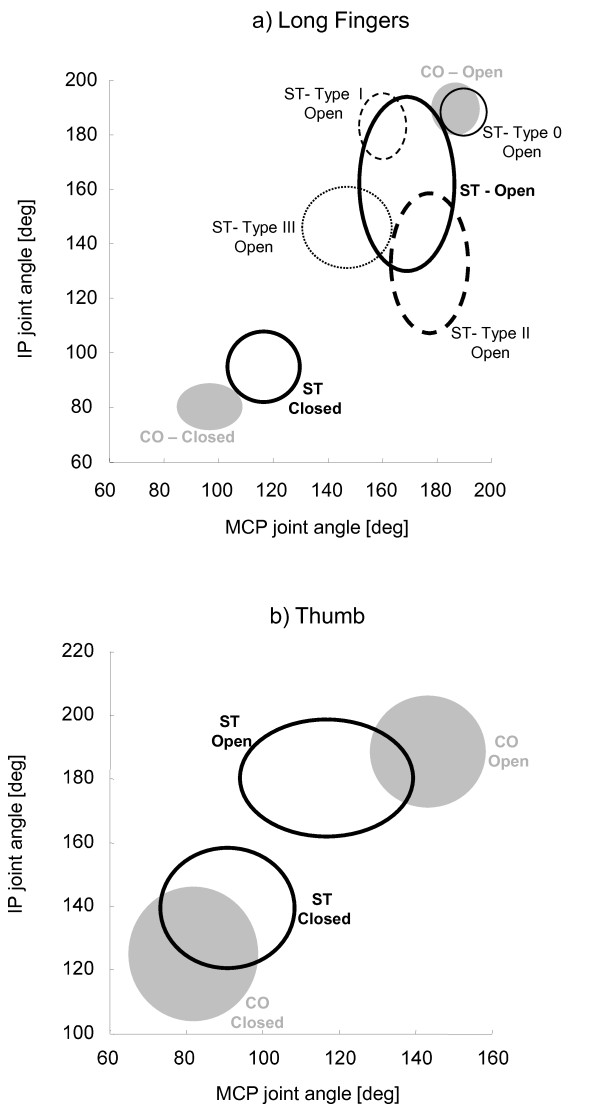
**Maximum flexion and extension angles**. Maximum extension angles (OPEN) and maximum flexion angles (CLOSED) of MCPJ and IPJ of long fingers (a) and thumb (b) for healthy subjects (CO) and stroke patients (ST). Confidence ellipsoids are shown for controls (grey) and for type 0, type I, type II and type III fingers of stroke subjects (black lines).

**Figure 8:**
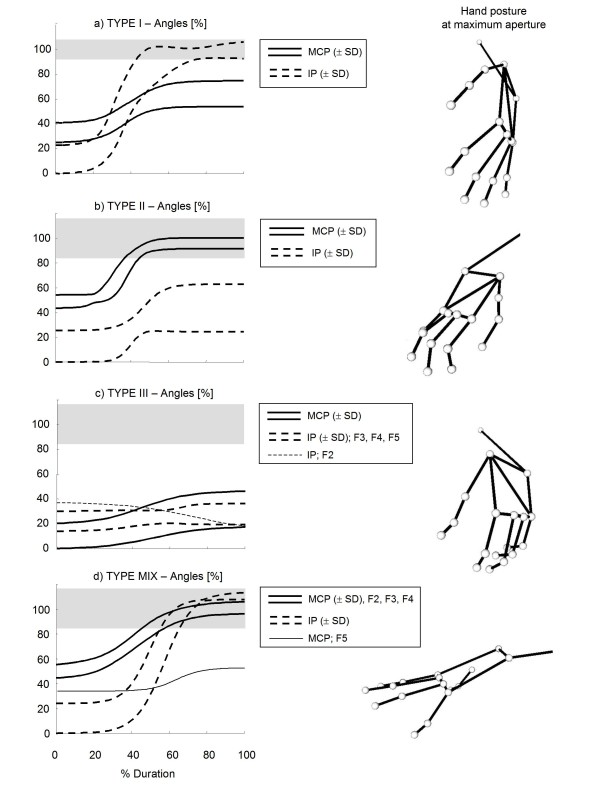
**Examples from stroke subjects**. Joint angles (± SD band) of four representative stroke subjects during hand opening (left panels) and corresponding snapshots of hand postures at maximum aperture (right panels). Grey bands represent healthy control range (± SD). To facilitate comparisons, angles are represented as a percentage of the range between the resting angle of the patient (0%) and the mean maximum extension angles of healthy subjects (100%). Type I hand (a) showed reduced extension of MCPJ and normal extension of IPJ. Type II hand (b) revealed reduced extension of IPJ and normal extension of MCPJ. Type III hand (c) showed reduced extension of both MCPJ and IPJ. Note that the subject's attempt to extend index IPJ (thin dashed line) resulted in an undesired flexion. Type MIX subject (d) showed different behaviour among long fingers. In particular, finger 2 to 4 revealed normal extension of both MCPJ and IPJ (type 0 fingers), while finger 5 (thin line) showed impairment of MCPJ only (type I finger).

Considering the high variability of maximum extension angles of long fingers' joints, represented by an inter-subject standard deviation 2 to 3 times greater than that of healthy controls (see Table 3 and Figure 7a), a more specific inspection of each digit was performed. This further analysis was based on the preliminary hypothesis that each finger would show, when hand is maximally open, one of the following four conditions: i) unaltered MCPJ and IPJ extension (type 0 finger); ii) reduced MCPJ extension and normal IPJ extension (type I); iii) reduced IPJ extension and normal MCPJ extension (type II), iv) reduction of both IPJ and MCPJ extension (type III). On the basis of this hypothesis, each hemiparetic hand could show either uniform involvement of all long fingers (type 0, type I, type II and type III hand), or differential impairment among digits (type MIX hand). Application of this scheme to the analysed sample of stroke subjects (see Table 4) revealed that one hand showed unaltered MCPJ and IPJ maximum angles (type 0), one hand showed nearly normal IPJ angles and reduced MCPJ extension (type I), three hands revealed an impairment mainly due to IPJ (type II), three hands showed a high reduction of both MCPJ and IPJ maximum extension angle (type III), while, in the remaining six hands (type MIX), long fingers showed characteristics different among each other, thus belonging to different types. Figure 7a depicts the angles of maximum extension (hand open) and maximum flexion (hand closed) for control subjects and each type of hemiparetic hand. Figure 8 depicts the examples of four stroke subjects showing type I, II, III and MIX hands. Contrarily to maximum extension angles, no differences among different hands were noticed in long finger angles at closed hand (see Figure 7a).

**Table 4:** Hand and finger type for all stroke subjects

Subject	Hand Type	Finger 2 Type	Finger 3 Type	Finger 4 Type	Finger 5 Type
ST1	**MIX**	I	I	0	0

ST2	**I**	I	I	I	I

ST3	**II**	II	II	II	II

ST4	**MIX**	0	0	0	I

ST5	**III**	III	III	III	III

ST6	**II**	II	II	II	II

ST7	**MIX**	I	I	III	III

ST8	**III**	III	III	III	III

ST9	**III**	III	III	III	III

ST10	**MIX**	II	II	I	I

ST11	**II**	II	II	II	II

ST12	**MIX**	0	0	I	I

ST13	**0**	0	0	0	0

ST14	**MIX**	II	II	II	I

Results related to maximum extension and maximum flexion angles of the thumb joints did not reveal any specific difference among hands (see Figure 7b). In particular, all thumbs showed a significant reduction of MCPJ maximum extension at hand open and a slight reduction of IPJ maximum flexion at hand closed.

#### Inter-joint and inter-digit coordination

**Figure 9:**
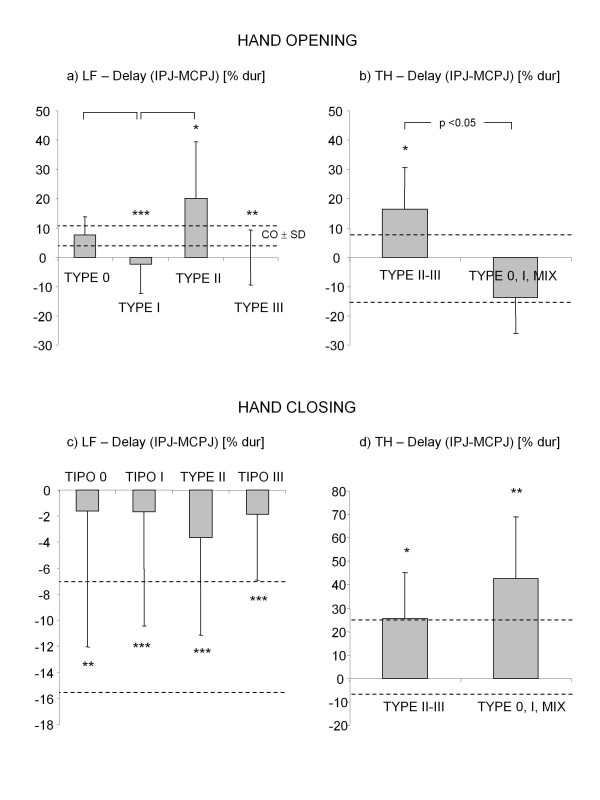
**Inter-joint coordination in stroke subjects**. Delay between IPJ and MCPJ of thumb (TH) and long fingers (LF) for stroke subjects, during hand opening (a, b) and hand closing (c, d). Columns and whiskers represent mean and standard deviation, respectively. Dashed horizontal lines represent healthy control range (± SD). *p < 0.05, **p < 0.01, ***p < 0.001 (Stroke Type vs Control, Mann-Whitney U test). Significant differences among stroke types are shown.

**Figure 10:**
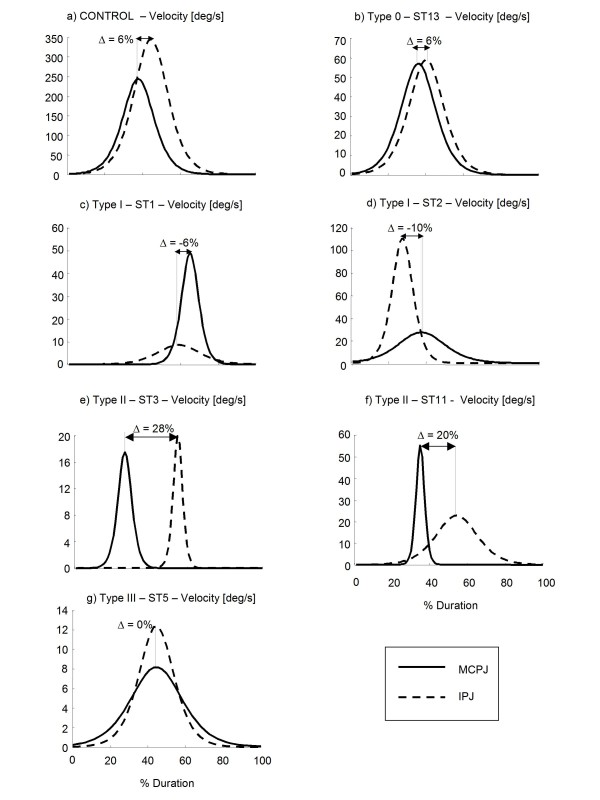
**Angular velocity during hand opening**. Velocity profiles of MCPJ and IPJ of a single finger of a representative healthy subject (a) and of six stroke patients (b, c, d, e, f, g) during hand opening. Delay (Δ) between IPJ and MCPJ angles are shown. Note that type 0 hand (b) showed a delay in the control range. Type I hands (c, d) revealed a reversed inter-joint coordination sequence (IPJ-MCPJ), while type II hands (e, f) showed prolonged delay with respect to controls. Type III hand (g) showed a contemporary extension of both MCPJ and IPJ.

Results related to IPJ-MCPJ delay revealed that the proximal-to-distal sequence typical of controls during hand opening was highly disrupted in stroke subjects. Figure 9a shows the mean (± SD) values of delay parameter for the different types of long fingers. Delay of type 0 digits (i.e. unaltered extension of both MCPJ and IPJ) was in the control range (see also Figure 10b). Type I fingers (i.e. impairment of MCPJ extension only) showed a negative average delay (Figure 9a) which indicated a reversed opening sequence (i.e. MCPJ followed by IPJ in reaching peak speed). This was caused, in 30% of the digits, by a delayed motion of MCPJ (Figure 10c), while in the remaining 70%, by a significantly slowed movement of MCPJ (Figure 10d). Type II digits (i.e. impairment of IPJ extension only) revealed a significantly higher delay with respect to healthy subjects (Figure 9a), which was due, in 30% of the cases, to a segmented movement in which IPJ started moving after MCPJ had already reached full extension (Figure 10e) and, in 70% of the cases, to a significantly slower movement of IPJ with respect to MCPJ (Figure 10f). In type III digits (i.e. impairment of both MCPJ and IPJ extension), MCPJ and IPJ contemporarily reached peak velocity as indicated by the delay value approximately equal to 0 (Figure 9a and Figure 10g). Impairment of inter-joint coordination was noticed also in the thumb of type II and type III hands which showed a reversed sequence of movement (MCPJ first followed by IPJ), as shown in Figure 9b. Inter-joint coordination was altered also during hand closing. Even though inter-digit variability was extremely high, mean values showed a reduced delay in long fingers, with MCPJ and IPJ which flexed almost synchronously (Figure 9c). Thumb, instead, revealed an abnormally high delay with respect to controls (see Figure 9d).

Stroke subjects showed a reduction of the inter-digit coordination indexes greater than 50% with respect to healthy controls. In particular, COI_LF _mean (± SD) value was 32.0% (± 26.8%) during hand opening and 45.2% (± 36.2%) during hand closing. The same trend was noticed after inclusion of the thumb, as reported by COI_HAND _values (hand opening: 48.1% ± 40.5%; hand closing: 34.9% ± 35.3%).

## Discussion

At present, there is still a lack of studies investigating the temporal features of hand movement and the inter-joint coordination aspects of multi-joint fingers motion in subjects with stroke. The present study focused on this aspect.

### Joint angle mathematical characterization and accuracy

The hyperbolic tangent function chosen for data modelling [18,24] was successful in characterizing the MCPJ and IPJ angular displacements of long fingers and thumb during hand opening and closing in healthy controls, thus confirming the results found by Braido and Zhang [18]. The model demonstrated a high level of accuracy also in the characterization of MCPJ and IPJ flexion/extension movements of stroke subjects (95% of movements). Only 5% of the MCPJ and IPJ angular profiles were not well fitted by the model. As shown in the example of Figure 4d, in these cases finger joints didn't show a monotonic sygmoidal-shape motion, but rather, a biphasic movement. In particular, the specific joint extended for approximately 50% of the cycle, reached maximal extension and than started flexing, probably because the subject was not able to maintain that level of extension for the whole movement duration.

As for thumb abduction angle (TAB), the mathematical model accurately characterized only 75% of the considered angular profiles, both in controls and in stroke subjects. This result revealed the existence of two sub-groups of subjects who adopted two different strategies in moving the thumb during hand opening. In the first sub-group thumb abduction and, consequently, thumb distance from the palm monotonically decreased during hand opening following a sygmoidal-shape profile (see Figure 4a). In the second sub-group (see Figure 4b) instead thumb started moving away from the palm, reached maximum abduction approximately at 50% of the movement and then started rotating towards the palm, thus reducing the abduction angle. This result could be ascribed to individual peculiarities of the subjects or to the fact that thumb position at hand maximally closed was not fixed during the experiment. Considering that the selected task was difficult to be performed by most stroke subjects, the subject was left free to execute the movement as best he could. The only given instruction was to close the hand, avoiding flexion of long fingers around the thumb. For this reason initial position of thumb in closure could have been on the radial side of the index finger or on its dorsal aspect: this variability in thumb initial posture could have influenced its movement during hand aperture.

With respect to other mathematical functions used in literature, the selected mathematical model has proven to be a good choice, as it needs the identification of a number of parameters (n = 4) lower than that required by the polynomial functions also used to characterise sygmoidal-shape movement profiles (n > = 6) [28]. Moreover, as noticed by Zhang and Chaffin [24], the four parameters used in the presented model are related to precise kinematic variables, while the parameters describing polynomial models hardly relate to any physical meaning.

### Test-retest reliability

Results related to test-retest reliability ascertained that the output generated by the model was highly repeatable, as indicated by the ICC values that were greater than 0.75 for all four parameters [12,26]. Mean absolute test-retest errors of the two angular parameters c_1 _and c_2 _were lower than 3.1° and thus lower than those defined for manual goniometry (between 7° and 9°), considered in clinical practice to be the gold standard of joint angle measurements [29]. Comparison with previous methods described in literature revealed mean absolute test-retest errors comparable to those reported by Dipietro et al [7] (6.2°), Degeorges et al [10] (8.0°), Carpinella et al [11] (7.3°) and Metcalf et al [12] (5.1°). Previously published research has not addressed the issue of reliability of the temporal parameters of hand movement. It was therefore not possible to compare the results of parameters c_3 _and c_4_.

Maximum test-retest errors, calculated as suggested by Bland & Altman [27], were lower than 7.2° for angular parameters and lower than 9.0%Dur for temporal parameters. As described in [27], these values could be used as indicators of the minimum significant change that can be detected by the method. It must be highlighted that the repeatability analysis was performed on unimpaired subjects only. Future study should extend this analysis also to stroke subjects.

Analysis of inter-session and intra-session standard deviations demonstrated that test-retest errors were mainly due to variation among repetitions in the same session (> 90% of the variability), rather than to variations among different test sessions (<10% of the variability). This could suggest that the markers repositioning procedure typical of test-retest sessions has a limited influence on data variability. Future studies should explore this aspect more deeply.

### Hand motion characterization in healthy subjects

In healthy subjects IPJ of long fingers showed, with respect to MCPJ, a greater ROM due to higher maximum flexion angles and higher peak velocity both in hand opening and closing. Analysis of the temporal aspects of hand motion revealed two typical inter-joint coordination patterns in hand opening and closing respectively. During hand opening, IPJ of the thumb started the movement followed by MCPJ_1_, while long fingers showed a typical proximal-to-distal sequence, with MCPJ which anticipated IPJ of approximately 7.4%Dur. These results confirmed those found by Somia et al [16] and Nakamura et al [17]. The presence of a stable coordination sequence between finger joints suggests the existence of a precise neurophysiological control mechanism in which, hypothetically, the extensor digitorum communis, that is the prime mover of long fingers' MCPJ, is the first muscle to be activated followed by lumbricals and interossei muscles (intrinsic muscles) that are the major extensors of IPJ [17]. During hand closing this coordination sequence appeared reversed confirming the results found by Somia et al [16]. In particular, long fingers IPJ and thumb MCPJ start flexing followed by thumb IPJ and long fingers MCPJ. This characteristic order of long fingers joint motion during hand closing (i.e. IPJ followed by MCPJ) has been previously explained with the presence of a significant activity of extensor digitorum communis also during finger flexion [30,31]. In this case the activation of the extensors would act as a brake on the MCPJ, thus resulting in movement initiation at the IPJ. These typical coordination patterns have been demonstrated to be stable among digits, as indicated by the synchronous movements of all MCPJ and IPJ which resulted in a IPJ-MCPJ delay not significantly different among long fingers. The simultaneous movement of joints of the same type was found also by Santello et al [19] during movements of reaching and grasping demonstrating a high level of inter-digit coordination in unimpaired hands.

### Hand motion characterization in stroke subjects

Results of the kinematic analysis demonstrated that the proposed method was able to strongly discriminate the motor performance of stroke sufferers from that of healthy subjects and to identify different types of hand dysfunction among hemiplegic subjects.

General analysis on the entire stroke group showed that, compared to healthy controls, patients took longer time to attain smaller angular displacements with significantly decreased peak velocities and a reduction of inter-digit coordination of more then 50% with respect to controls. These impairments were present in both hand opening and closing.

#### Hand opening in stroke

Maximum extension angles were significantly lower in all joints, with respect to controls (p < 0.001). Deficit of finger extension has been demonstrated to be the results of two concurrent causes: mechanical restraint to extension and altered neurophysiological control mechanisms. A number of studies have documented changes in the mechanical properties of upper limb muscles. In particular, atrophy of extensors [32] and contractures of flexors caused by shortening of muscle fibres and increased passive stiffness of muscular tissue [23] have been demonstrated to contribute to limit fingers extension. However, deficit in hand opening has been documented also in stroke subjects who didn't present with increased passive resistance [33], suggesting that anomalies in neurological control play a major role in reducing finger joints motion. Three main neuromotor causes have been demonstrated to interfere with hand opening. The first alteration is flexors spasticity, an involuntary velocity-dependent contraction of flexor muscles during finger extension due to an exaggerated stretch reflex activity [33,34]. The other two aspects are excessive co-contraction of flexors and extensors [5,35] and weakness of extensor muscles, presumably caused by a reduction in the activation of spinal segmental neurons [36].

Inspection of each stroke subject, revealed the existence of four different behaviours of the hemiparetic hand during opening. Of fourteen hands analysed, one was almost unaltered (type 0 hand), seven had uniform involvement of all long fingers (type I, type II and type III hands), while six showed differential impairment among digits (type MIX hands). Type I fingers showed a nearly normal motion of IPJ and a reduced extension of MCPJ, associated with a reverse inter-joint coordination sequence (i.e. distal-to-proximal). As reported by Kamper et al [35], the weakness of extrinsic extensors (i.e. extensor digitorum communis) and the exaggerated co-contraction of extrinsic flexors (i.e. flexor digitorum profundus) could justify the reduced motion of MCPJ, while a good activation of intrinsic muscles (interossei and lumbricals) could explain the physiological extension of IPJ. The reversed distal-to-proximal synergy has been demonstrated to be partly due to a delayed motion of MCPJ (see Figure 10c) possibly explained by an abnormally high brake action of extrinsic flexors [30], and partly caused by a significantly slower movement of MCPJ (see Figure 10d) possibly due to slow and weak activation of extensor digitorum communis. Contrarily to type I, type II digits revealed impairment of IPJ extension only, with a significantly high delay between IPJ and MCPJ in long fingers. This pattern of movement appeared similar to the task of voluntary curling the fingers while extending MCPJ, described by Long & Brown [30] in healthy controls. During this task, the authors reported the co-activation of extensor digitorum communis and flexor digitorum profundus, with silent activity of lumbricals and interossei (prime extensors of IPJ). From this comparison, it can be speculated that type II fingers could show a physiological activation of extensor digitorum communis, an abnormally high co-activation of extrinsic flexors and a severe weakness of intrinsic muscles (lumbricals and interossei), which in turn, would explain the unimpaired movement of MCPJ and the reduced extension of IPJ. The high IPJ-MCPJ delay has been demonstrated to be due, in 30% of the cases, to a segmented movement in which IPJ start moving after MCPJ has already reached full extension (see Figure 10e) and, in 70% of the cases, to an abnormal slowness of IPJ in completing the movement (see Figure 10f). In the first case the high value of parameter Δ could be caused by a delayed but fast activation of lumbricals which generates a stretch reflex on IPJ flexors, while, in the second case it could be explained mainly by lumbrical weakness and slow prolonged activation, rather than to a delayed reclutation of muscle fibers. Finally, the most impaired hands (type III), which revealed reduction of both MCPJ and IPJ extension, possibly show all the muscle activity anomalies described for type I and type II hands.

In three cases, subjects attempts to open their hand resulted in an inappropriate flexion of one or two IPJs of the hand, as found also by Kamper et al [35]. Again, the origin of this anomalous behaviour could be ascribed to the exaggerated co-activation of flexor muscles, possibly due to the loss of descending inputs involved in reciprocal inhibition of flexor muscles [37] and/or to a preferential activation of cortical neurons responsible for co-contraction of antagonists muscles [35].

Thumb extension was impaired in all subjects. Inter-joint coordination pattern was preserved, with the exception of type II and type III hands which showed a reversed inter-joint sequence and significantly high delay of IPJ, possibly due to an inversion of the activation of extensor pollicis longus and brevis.

#### Hand closing in stroke

Maximum flexion was significantly reduced in all joints, thus indicating anomalies not only in hand opening but also in hand closing. However, peak speed reached during hand closing was significantly higher than that obtained during hand opening, thus confirming that finger flexion was less impaired than finger extension as reported in literature [5]. Considering that spasticity of finger extensors was rarely observed in stroke subjects [33], impairment in hand closing could be ascribed to flexors weakness well documented in literature [5,36]. Contrarily to hand opening, hand closing didn't reveal differences among different hand types. All hands showed a similar inter-joint coordination sequence which is maintained (i.e. IPJ first followed by MCPJ) though impaired as demonstrated by the significantly reduced inter-joint delay. A possible explanation of the almost contemporary flexion of MCPJ and IPJ could be found in the study of Darling et al [31]. The authors observed that in some healthy subjects activity of interossei muscles was consistently present during finger flexion. It could be that the co-activation of the intrinsic extensors is increased in stroke subjects, thus producing a brake to IPJ delaying their flexion movement. A similar speculation could be made to explain the high delay between IPJ and MCPJ of the thumb: a possible activity of the extensor pollicis longus during hand closing could oppose IPJ, thus delaying its flexion. Future studies on the electromyographic activity of hand muscles are required to confirm the hypothesis made in this work to explain hemiparetic hand impairments.

### Limitation of the study

There are some limitations that need to be addressed regarding the present study.

A first limitation is the small number of hemiparetic subjects included in the protocol. The proposed evaluation method should be tested on a greater number of patients in order to make the results generalizable to the entire population with stroke. Also, a second study testing both the involved and the non-involved hand of the person with hemiparesis might be indicated in order to compare coordination patterns within subject.

The second limitation concerns thumb angles calculation. In particular MCPJ_1 _and TAB angles, as computed in the present study, describe the movement of the thumb's proximal phalanx with respect to the metacarpal plane of the hand, which involves the motion of two joints, i.e. metacarpophalangeal (MCPJ) and trapeziometacarpal (TMCJ) joints, and four degrees of freedom. For this reason the angles computed in this work do not provide an accurate characterization of MCPJ and TMCJ motion but rather describe the time-course of thumb orientation with respect to the palm, which was considered more relevant for the topic of the present study. It is possible that this simplified characterization of thumb kinematics is correlated to the difficulty of the chosen mathematical model to accurately describe thumb motion.

A third potential limitation is related to the time required for the testing session. Optoelectronic motion-analysis requires more expensive instrumentation and more time-demanding setting-up procedures with respect to lower-cost sensorized gloves, presently used to evaluate unimpaired individuals [19] and stroke subjects with mild hand motor impairment [14,38]. On the other hand, as reported by Simone & Kamper [39], the existing glove systems are often difficult to don and remove for individuals with severe hand disorders and they could further reduce sensory inputs, already impaired in stoke patients [40], thus worsening hand motor performances. For these reasons an optoelectronic motion analyser, which allows the execution of the experiments in a more ecological context, was chosen, also considering that, in the last years, this kind of systems are increasingly included in clinical instrumentation.

## Conclusions

The quantitative method proposed in the present study has been demonstrated to be a valid tool to i) accurately characterise hand opening/closing movements in healthy subjects and persons with hemiparesis due to stroke ii) objectively evaluate changes of performance with an adequate sensitivity provided by low test-retest errors, iii) quantify hemiparetic hand motor deficits and discriminate motor performances of stroke sufferers from those of healthy controls. Correlation of the present results with electromyographic data and clinical tests related to hand function and lesion localization will be warranted to evaluate the efficacy of the proposed method to predict the potential of motor recovery and to plan rehabilitation treatments tailored to the specific hand deficit of each person with stroke.

## Competing interests

The authors declare that they have no competing interests.

## Authors' contributions

The overall design of the experiment was agreed by all authors after extensive discussions. JJ selected the subjects and conducted the clinical evaluations. IC and JJ participated in data acquisition. IC analysed the data, performed the statistical analysis and performed data interpretation. JJ and MF participated in data interpretation. IC wrote the manuscript. JJ and MF reviewed the manuscript. All authors read and approved the final manuscript.

## Note

For example, if c_*3 *_= 0.45 and c_*4 *_= 0.2, then c_*2 *_= .[*α*_*e*_*(ΔT)- α*_*e*_*(0)*]/[tanh(2.75) + tanh (2.25)]~ [*α*_*e*_*(ΔT)- α*_*e*_*(0)*]/2.
